# Cellulose Nanocrystals Loaded with Thiamethoxam: Fabrication, Characterization, and Evaluation of Insecticidal Activity against *Phenacoccus solenopsis* Tinsley (Hemiptera: Pseudococcidae)

**DOI:** 10.3390/nano10040788

**Published:** 2020-04-20

**Authors:** Asem Elabasy, Ali Shoaib, Muhammad Waqas, Zuhua Shi, Mingxing Jiang

**Affiliations:** 1 Key Laboratory of Molecular Biology of Crop Pathogens and Insects, Ministry of Agriculture, Institute of Insect Sciences, College of Agriculture and Biotechnology, Zhejiang University, Hangzhou 310058, China; asemelabasy@hotmail.com (A.E.); alizaky66@hotmail.com (A.S.); muhammadsaad@zju.edu.cn (M.W.); zhshi@zju.edu.cn (Z.S.); 2 Department of Pesticides, Plant Protection Research Institute, Agricultural Research Center, Cairo 11341, Egypt

**Keywords:** cellulose nanocrystals, thiamethoxam, insecticidal activity, *Phenacoccus solenopsis*

## Abstract

Using smart nanopesticide formulations based on nanomaterials can offer promising potential applications for decreasing pesticide residues and their effects on human health and the environment. In this study, a novel nanoformulation (NF) of thiamethoxam (TMX) was fabricated using the solvent evaporation method through loading TMX on cellulose nanocrystals (CNCs) as the carrier. The synthesized TMX-CNCs was investigated through different techniques, such as Fourier transform infrared spectrometer (FT-IR), X-ray diffraction (XRD), transmission electron microscopy (TEM), dynamic light scattering (DLS), and thermogravimetric analysis (TGA). The results revealed that the loading efficiency and entrapment efficiency were 18.7% and 83.7 ± 1.8% for TMX, respectively. The prepared nanoformulation (TMX-CNCs) had a width of 7–14 nm and a length of 85–214 nm with a zeta potential of −23.6 ± 0.3 mV. The drug release behavior study exhibited that the release of TMX from TMX-loaded CNCs was good and sustained. Furthermore, bioassay results showed that the insecticidal activity of TMX-CNCs against *Phenacoccus solenopsis* was significantly superior to that of the technical and commercial formulation, as indicated by the lower LC_50_ value. The results indicate that the TMX nanoformulation has great potential for application in agriculture for pest control.

## 1. Introduction

Pesticides play an indispensable role in agriculture and are needed for crop protection against insect pests, weeds, and plant pathogens [1]. According to the data from the United Nations Food and Agriculture Organization (FAO), pesticides can save 30% of the total crop production losses worldwide [2,3]. However, they also cause several adverse impacts on our life and ecosystems because of their toxicity to humans and non-target organisms [4]. Unfortunately, depending on the modes of application and environmental conditions, over 90% of the traditional pesticide formulations are lost or decomposed owing to degradation, evaporation, leaching, and runoff during field application, and only ~0.1% of the pesticide can ultimately affect harmful target pests [1,5,6,7]. In order to compensate for these losses and to maintain effective control of pests, conventional pesticide formulations are often applied at concentrations that are much higher than that desired to reach the required effect [8,9]. Consequently, the intensive and irrational usage of chemical pesticides not only increases agrochemicals’ economic costs, but also adversely affects the environment and non-target organisms [10]. Therefore, it is a crucial need to develop new pesticide formulation technologies to enhance the efficacy of pesticide use, and thereby decrease environmental risk [11].

The substantial advancement of nanotechnology and nanomaterials has paved the way in recent years for the creation of new pesticide formulations that are less environmentally damaging, cost-effective, and more efficient as compared to traditional formulations [12]. Nano-based pesticide formulations might bring useful enhancements in the characteristics and behavior of pesticides like solubility, dispersion, stability, and targeting delivery. Moreover, it can improve the pesticide utilization efficiency, prolong the effective duration, minimize pesticide loss by decreasing runoff into the environment, protect active ingredients against premature degradation, reducing the dosage needed, and avoid the use of harmful organic solvents [1,13]. Additionally, nanopesticide delivery systems have better adhesion to crop leaf surfaces, and allow the delivery and release of active ingredients to target sites [14,15,16]. In this direction, many kinds of nanomaterials are developed and used in agricultural systems, such as silica, metal, metal oxides, lipids, carbon, and polymeric nanoparticles [17,18,19], to carry numerous variety of agrichemicals, involving insecticides, herbicides, fungicides, and fertilizers, because they are low cost, non-toxic, eco-friendly, and effective at lower doses [20,21]. Among these, cellulose nanocrystals (CNCs) have received significant attention in recent years, due to their unique physical properties, high surface area, biodegradability, biocompatibility, favorable chemical modification, and low cytotoxicity [22]. These remarkable features make CNCs a promising candidate for many applications in different fields, such as biomedical engineering [23], drug carriers [24], electronics, catalysis, and Pickering emulsifier [25,26,27]. However, there are limited studies about using CNCs as nano-carrier in the field of pesticide. Tang et al. [28] developed a new approach to the preparation of templated polydopamine (PDA) microcapsules using stabilized Pickering emulsions through cinnamoyl chloride modified cellulose nanocrystals for essential oil and the encapsulation of pesticides. 

**Figure 1:**
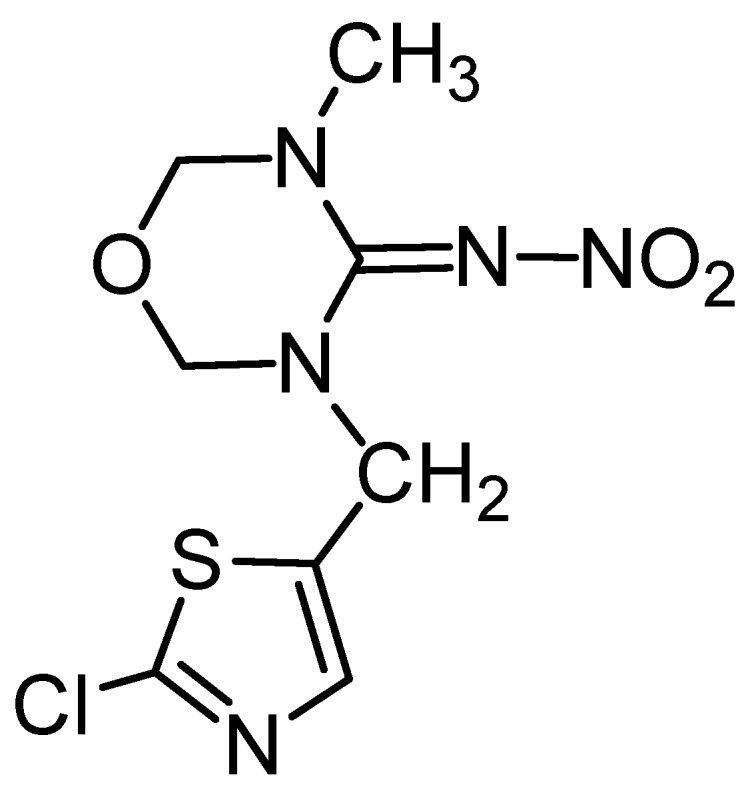
Chemical structure of Thiamethoxam.

Thiamethoxam (TMX) (3-(2-chloro-1,3-thiazol-5-ylmethyl)-5-methyl-1,3,5-oxadiazinan-4-ylidene(nitro)amine, Figure 1) is a fast-acting systemic insecticide being used extensively around the world, belonging to the neonicotinoids group. TMX is a highly effective insecticide with a broad spectrum against sucking pests like whiteflies, aphids, and mealybugs. It acts as an agonist through binding to insect nicotinic acetylcholine receptors in the central nervous system [29,30,31]. Currently, the formulations of TMX available in the market are WG, SC, FS, and WS. Gupta et al. [32] studied the leaching and dissipation behavior of conventional TMX formulations, and found that these commercial formulations can leach under heavy rainfall conditions. Moreover, the main problem associated with TMX is its high aqueous solubility [32], which allows it to reach the natural water resources and causes adverse effects on the environment. Developing novel formulations to minimize the harmful effects, as mentioned above, and reduce the associated risk through controlled release formulation technology is therefore urgently needed. 

In the present study, CNCs were used to fabricate TMX nanoformulation by using the emulsion solvent evaporation method. The synthesized nanoformulation containing TMX was characterized by Fourier transform infrared spectrometer (FT-IR), X-ray Diffraction (XRD), transmission electron microscopy (TEM), dynamic light scattering technique (DLS), thermogravimetric analysis (TGA), and UV-vis spectrophotometry. The entrapment efficiency (EE), loading capacity, and controlled release were also investigated. Moreover, bioassays against the cotton mealybug, *Phenacoccus solenopsis* Tinsley were conducted to assess the insecticidal activity of nanoformulation and compared with technical grade and commercial formulation. CNCs are employed as carrier systems for pharmaceuticals [33]. Our aim of this work was to develop a novel formulation for TMX using CNCs as a carrier, because its biodegradability can not only help to reduce environmental influences, but also contribute to the controlled release of the pesticides.

## 2. Materials and Methods 

### 2.1. Materials

The model pesticide, TMX (97%) was purchased from Shanghai Bosman Industrial Co., Ltd. (Shanghai, China). The commercial thiamethoxam water dispersible granule (TMX-WDG, 25%) was obtained from Syngenta Crop Protection Co., Ltd. (Jiangmen, China). Microcrystalline cellulose (MCC, 36 µm) and sulfuric acid (H_2_SO_4_) were purchased from Sangon Biotech Co., Ltd., (Shanghai, China). Poly(vinyl alcohol) (PVA) with the hydrolysis of 87–90% and an average molecular weight of 30–70 kDa was obtained from Aladdin Biochemical Technology Co., Ltd. (Shanghai, China). Acetonitrile and methylene chloride were procured from Sigma-Aldrich (St. Louis, MO, USA). Distilled water used in this study was obtained from the Milli-Q water purification system (18.2 MΩ cm, TOC ≤ 4 ppb). 

#### Insect Culture

Cotton mealybug, *P. solenopsis* was collected from ornamental plants in the eastern suburbs of Hangzhou, China, in June 2017. The cotton mealybug population was reared on tomato plants in cages and was maintained at 27 ± 2 °C and 65 ± 5% R.H. with a 14:10 (L:D) photoperiod in the laboratory of Insect Ecology and IPM, Institute of Insect Sciences, Zhejiang University (Hangzhou, China).

### 2.2. Preparation of TMX-NF

#### 2.2.1. Preparation of CNCs

CNCs were synthesized by acid hydrolysis method, as described by Beck-Candanedo et al. [34]. Ten grams of MCC was added into 100 mL 64% sulfuric acid (H_2_SO_4_) solution (w:w) and hydrolyzed at 45 °C for 1 h under vigorous magnetic stirring. The acid hydrolysis was stopped by diluting the reaction 10-fold with chilled distilled water. The hydrolysis solution was centrifuged at (Centrifuge 5810 R; Eppendorf, Hamburg, Germany) 11,000 rpm (12,851× *g*) for 15 min. The precipitates were washed four times with distilled water to decrease acid concentration, and then resuspended in distilled water and dialyzed until the pH reached 7.0. The sample was sonicated at 30% amplitude in an ice bath for 10 min to avoid overheating and then freeze-dried using a machine (Alpha 1-2 LD plus; Martin Christ Gefriertrocknungsanlagen GmbH, Osterode am Harz, Germany) to get CNCs powder.

#### 2.2.2. Preparation of TMX-Loaded CNCs 

**Figure 2:**
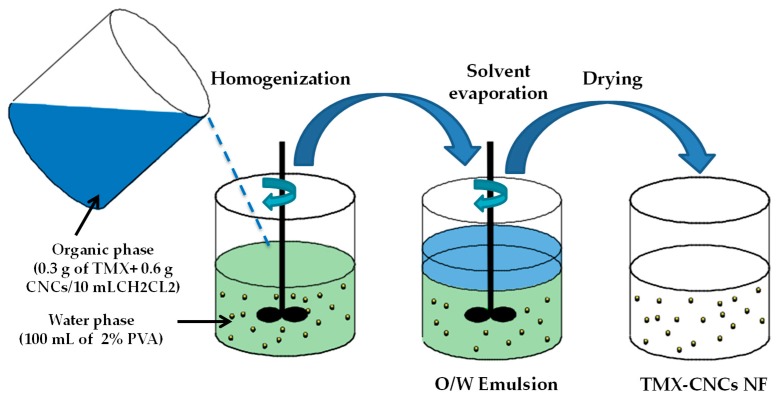
Schematic description of the preparation of the thiamethoxam (TMX)-loaded cellulose nanocrystals (CNCs).

TMX-loaded CNCs were synthesized using an oil-in-water (O/W) emulsion solvent evaporation method, according to Zhang et al. [35] and Elabasy et al. [36]. Briefly, the aqueous phase was prepared by dissolving PVA (2% w/v) in water as an aqueous solution, whereas the organic phase was obtained by mixing 0.3 g of TMX and 0.6 g of CNCs in methylene chloride (10 mL) in an ice-water bath. Afterwards, the organic phase was added to the aqueous phase dropwise while stirring by a homogenizer (T18 digital Ultra-Turrax, IKA, Staufen, Germany) at 10,000 rpm for 10 min to generate a stabilized O/W emulsion. The residual organic solvent in the water solution of the O/W emulsion was evaporated with constant stirring at 700 rpm and 30 °C overnight. The prepared TMX-CNCs were collected by centrifugation (Centrifuge 5417 R; Eppendorf, Hamburg, Germany) at 10,000 rpm (10,621× *g*) for 10 min at 4 °C, washed thrice with distilled water, and then lyophilized to obtain a free-flowing powder. The synthetic route of TMX-loaded CNCs is schematically illustrated in Figure 2.

### 2.3. Characterization of CNCs and TMX-CNCs 

In order to identify the different functional groups present in the samples, a Fourier transform infrared spectrometer (FT-IR) (Vector 22, Bruker, Ettlingen, Germany) was used. The FT-IR spectra of CNCs and TMX were recorded at a resolution of 4 cm^−1^ in the range of 400–4000 cm^−1^. X-ray diffraction (XRD) was used to describe the structure of the prepared nanoparticles using the X’PERT-PRO-PANalytical apparatus (PANalytical, Almelo, The Netherlands) with Cu-K*α* radiation (*λ* = 0.15406 nm). The diffraction results were recorded at the 2*θ* angle with a resolution of 0.02° in the range of 5–80°. The crystallinity index (CI) of dried CNCs was calculated based on the ratio of the crystalline peak (200) to the total area under the amorphous curve. A JEM-1230 transmission electron microscopy (TEM, JEOL, Akishima, Japan) was used to study and characterize the samples’ structure and particle size. The samples were prepared in distilled water via dispersion, and a drop of the diluted solution was placed onto a carbon-coated copper grid, and then allowed to dry at room temperature. The length was determined through a straight line between the two ends of the crystal. The width at the midpoint was measured unless a particle was asymmetric, in which case the widest point was measured. Such measurements were made for CNCs and TMX-CNCs by software named Image-Pro Plus (version 6.0) from the TEM images of more than 50 nanocrystals. A Zetasizer Nano ZS90 Analyzer (Malvern Instruments Ltd., Malvern, UK) was used to measure the particle size, polydispersity index, and zeta potential. The average value was reported, and each sample was measured three times. The data were processed using the cumulants analysis method in the Malvern software. Thermogravimetric analysis (TGA) was carried out to determine the loading efficiency of TMX by using an SDT Q600 (TA Instruments-Waters LLC, New Castle, DE, USA) apparatus from 25 to 800 °C with a heating rate of 10 °C/ min under nitrogen atmospheres. 

### 2.4. Determination of Entrapment Efficiency of TMX in the NF

The amount of TMX in nanoformulation was calculated through the difference between the total quantity of TMX added and the amount of TMX, which was unloaded with CNCs. The centrifuge was used to determine the entrapment efficiency (EE) of TMX in the nanoformulation. The TMX loaded with CNCs was centrifuged for 30 min at 15,000 rpm (23,897× *g*). The concentration of free TMX in the supernatant was determined by UV–vis spectrophotometer (UV-2600, Shimadzu, Kyoto, Japan) at 254 nm [37]. A blank sample was also prepared by the same method. The absorbance of samples was converted to concentration by using the standard curve. The entrapment efficiency of TMX was calculated as follow:

(1) $$EE\;\left(\%\right)=\frac{A_{total}-B_{free}}{A_{total}}\times 100$$

where *A_total_* is the total amount of TMX used to prepare nanoformulation and *B_free_* is the amount of free TMX in the supernatant.

### 2.5. In Vitro Release of TMX

The extent of TMX release from nanoformulation was conducted according to the literature method with some modification [38]. Briefly, an accurately weighed amount of the nanoformulation (10 mg) was added to the glass vial containing 20 mL of phosphate buffer solution (PBS; pH 7.4) as the release media and shaken at 100 rpm. At specific time intervals, 4 mL was withdrawn, replaced with 4 mL of fresh medium, and centrifuged at 10,000 rpm (10,621× *g*) for 20 min to obtain clear supernatant. TMX concentration in the supernatant was analyzed by monitoring its absorbance at 254 nm using UV–vis spectrophotometer. 

### 2.6. Bioassay

Bioassays of the TMX-NF were carried out using the method of leaf dipping against second instar nymphs of *P. solenopsis* [39]. Tomato leaves were immersed into different concentrations of TMX-NF, technical grade, and commercial TMX 25% WDG for 20 s and left to dry for 1.5 h at room temperature. A dried leaf was put into each petri dish (5 cm in diameter) with a piece of moist filter paper that was utilized to prevent the dryness of the leaves. Ten *P. solenopsis* second-instar nymphs were introduced into each petri dish, and each concentration was repeated thrice. For control, leaves were treated with water only. All bioassay was performed under the same laboratory conditions as mentioned above. The mortality was assessed 24, 48, and 72 h after exposure to various concentrations of TMX-CNCs, technical grade, and commercial TMX 25% WDG. Cotton mealybug nymphs were deemed dead if they did not show any movement of the leg when gently touched by camel’s hairbrush.

### 2.7. Statistical Analysis

Bioassay data were analyzed by using probit analysis [40] with POLO Plus software (version 2.0, LeOra Software, Berkeley, CA, USA) [41] to estimate LC_50_ values, 95% confidence limits (CLs), slope, and Chi-square (*χ*^2^). 

## 3. Results and Discussion

### 3.1. Synthesis and Characterization of TMX-NF

There has recently been a great deal of effort to develop pesticide delivery systems by using various materials. These materials include polymers, such as CNCs, which, because of their superior properties and potential applications in smart delivery systems, have become popular and widely used among nanomaterials. This study aims to develop CNCs as a carrier system for the TMX insecticide, which offers an alternative technique for pest control. Here, we prepared novel TMX nanoformulation, TMX-CNCs, based on the emulsion solvent evaporation technique. To generate a stabilized O/W emulsion, we emulsified the organic phase into the water phase using a high-speed disperser, and obtained the TMX-CNCs sample after evaporating the organic solvent. Such a method, besides the capacity of encapsulating hydrophilic and hydrophobic molecules, has also been used to improve drug loading and entrapment efficiency [12]. In our study reported here, the EE of TMX-CNCs formulation reached 83.7 ± 1.8% (Table 1). Taking together the results of our previous study [36], we suggest that CNCs-based nanoformulation has great potential for achieving higher pest-control efficacy, thereby decreasing costs, delaying insect-resistance development, and reducing side effects on the environment and human health. Regarding TMX encapsulation, challenges exist as there are limited reports of this procedure in literature [12]. Using beeswax as wrapping matrix (Thiamethoxam/beeswax-kaolin) microcapsules, Huang et al. [4] developed two types of Thiamethoxam, which had an entrapment efficiency of 82.0% and 72.1%, respectively.

#### 3.1.1. FT-IR Spectroscopy

**Figure 3:**
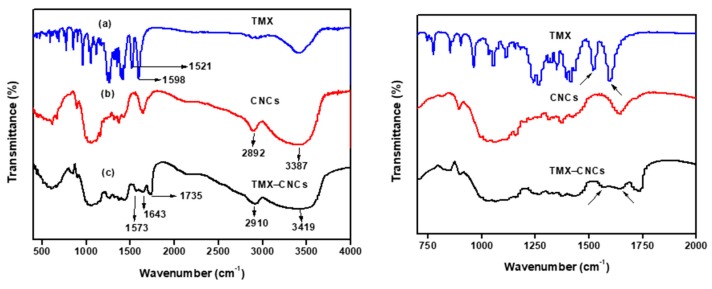
Fourier transform infrared (FT-IR) spectra of (**a**) TMX, (**b**) CNCs, and (**c**) nanoformulation (TMX-CNCs).

The FT-IR spectra of technical TMX, CNCs_,_ and TMX-CNCs are shown in Figure 3a–c. As shown in Figure 3b, the peaks that were observed at 3387 cm^−1^ (O–H stretching vibration), 2892 cm^−1^ (symmetric stretching vibration of C–H), 1645 cm^−1^ (originated from the absorbed water), 1418, 1373, and 1316 cm^−1^ (bending vibration of CH, CH_2_, OH, respectively), 1160 cm^−1^ (C–O–C asymmetric vibration), 1062 and 895 cm^−1^ (C–O stretching of the pyranose ring skeleton and the glycosidic linkages between glucose units in cellulose) belong to CNCs, respectively [42,43,44]. In the case of TMX, the strong peaks appeared at 1598 and 1521 cm^−1^ in the TMX spectrum (Figure 3a), which correspond to C=N and NO_2_ stretching frequencies, respectively [4,45,46]. The TMX-loaded CNCs spectrum (Figure 3c) shows most of the major characteristic bands corresponding to CNCs and TMX. The peak at 3419 cm^−1^ became broader in TMX-CNCs, suggesting increased hydrogen bonds between CNCs and TMX. The presence of the TMX in the CNCs was confirmed by a shift in peaks from 1598 to 1643 cm^−1^ and 1521 to 1573 cm^−1^, corresponding to C=N and NO_2_ bonds of the TMX. Compared to (Figure 3b), a new peak has emerged in the TMX-CNCs (Figure 3c) at 1735 cm^−1^, which can be ascribed to C=O group of the PVA. This result suggests that the TMX was successfully loaded into CNCs. 

#### 3.1.2. X-ray Diffraction Analysis

**Figure 4:**
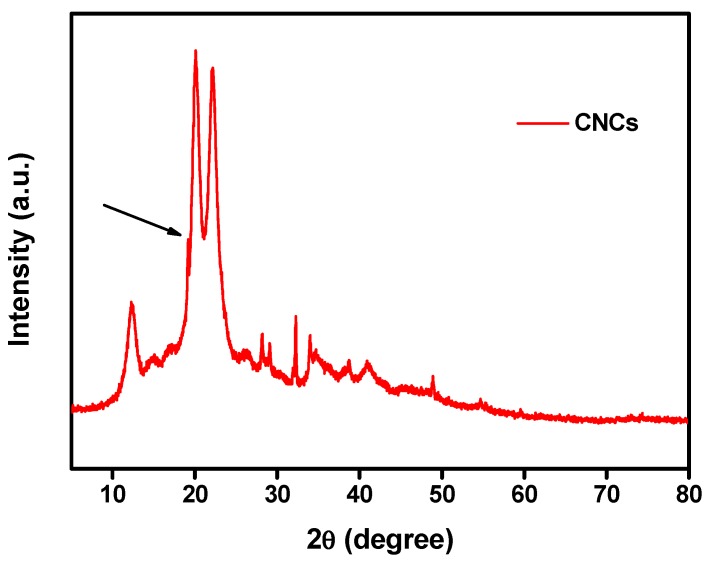
X-ray Diffraction (XRD) patterns of CNCs.

The crystalline structure nature of the synthesized CNCs was investigated based on the X-ray diffraction analysis, which revealed that there were three emission peaks at around 2*θ* = 12.0°, 20.1° and 22.1°, which correspond to the crystal planes (110), (210), and (200), respectively (Figure 4). After chemical treatments, the presence of crystalline peak as a doublet, as seen in Figure 4, which reflects the transformation of native cellulose from cellulose I to cellulose II that confirms CNCs formation [47]. The crystallinity index (CI) was calculated and found to be 71%. This result agreed with the earlier study [44].

#### 3.1.3. TEM Analysis

**Figure 5:**
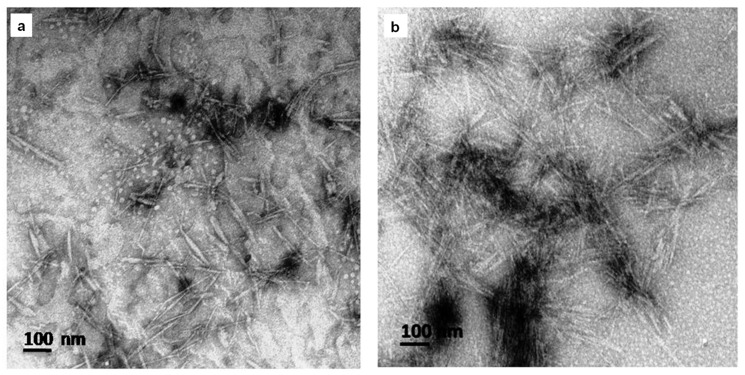
Transmission electron microscopy (TEM) images of (**a**) CNCs and (**b**) TMX-CNCs.

TEM was used to determine the structure and morphological shape of the synthesized CNCs and TMX-loaded CNCs (Figure 5). As seen in Figure 5a,b, TEM micrographs of CNCs and TMX-loaded CNCs represent the rod-like shape; however, there were some nanocrystals agglomerated. In addition, the width of the CNCs and TMX-CNCs were between 4–9 ± 1.7 and 7–14 ± 2.2 nm, and length between 68–206 ± 31.5 and 85–214 ± 54.3 nm, respectively. The agglomeration is generally resulted from the Van der Waals attraction forces between the nanoparticles, the water evaporation step and freeze-drying process, and strong inter-particle hydrogen bonding. The results were in line with the data obtained from many earlier reports [48,49,50].

#### 3.1.4. Particle Size and Zeta Potential

The dynamic light scattering technique (DLS) was conducted to examine the mean particle size, polydispersity index, and zeta potential (ζ), and the results are summarized in Table 1. The average diameter of the TMX-CNCs increased to 798.0 ± 149 nm, due to the loading TMX onto CNCs, while the polydispersity index (PdI) value of the CNCs was smaller than that of the TMX-CNCs (Table 1). The DLS particle size was larger than that measured by TEM. The discrepancy in particle size between DLS and TEM measurements may be explained through the fact that the DLS method measures the hydrodynamic layers surrounding the hydrophilic particles, or the agglomeration of single particles when dispersed in water, leading to an overestimation of particle size [51]. In contrast, TEM measures the actual diameter in the dry state. Moreover, a particle size distribution was homogeneous and monodispersed, as confirmed by the low PdI value except for TMX-CNCs. A PdI value (less than 0.5) suggests that a narrow and suitable particle size distribution in colloidal suspension. 

Zeta potential is a valuable tool for predicting nanomaterials’ stability in aqueous media. The ζ potentials values of CNCs and TMX-CNCs were −39.0 ± 1.8 and −23.6 ± 0.3 mV, as given in Table 1. The results indicate that TMX loading had led to a reduction in ζ potential in the case of TMX-CNCs. The negative ζ potential of the TMX-CNCs was because of the presence of OSO_3_ groups in the CNCs [50]. Based on the above results, the nanoformulation showed a good colloidal stability in aqueous solution in the current study. 

#### 3.1.5. Thermogravimetric Analysis (TGA)

**Figure 6:**
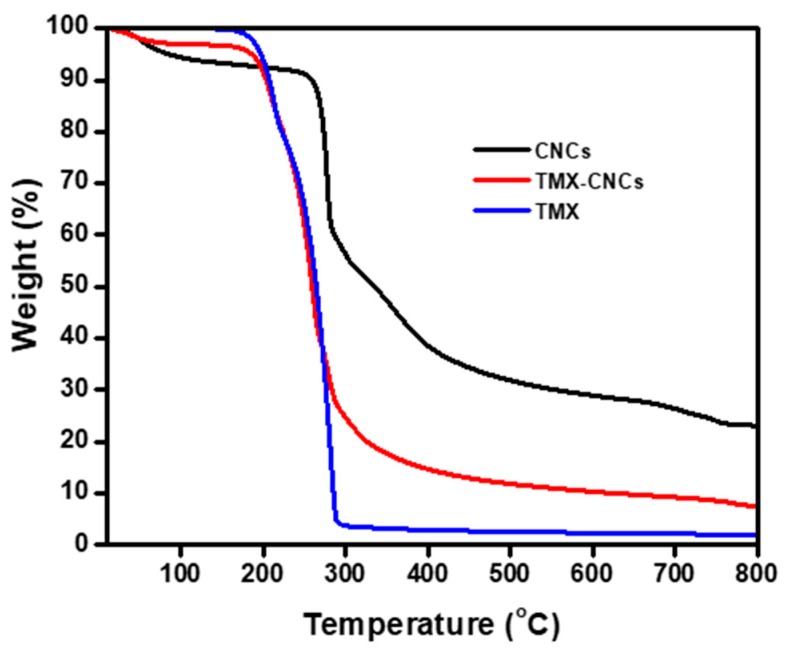
Thermogravimetric Analysis (TGA) curves for CNCs, TMX-CNCs and TMX.

TGA was used to investigate the drug loading efficiency and decomposition of materials. Figure 6 shows the TGA thermograms of CNCs, TMX-CNCs, and technical TMX. The TGA results displayed that the decomposition of CNCs starts at 316 °C, and the TMX decomposition starts at 212 °C. The weight loss before 200 °C might be resulted from evaporation in the samples. The weight loss between 212–285 °C could be assigned to the evaporation and decomposition of TMX, while the weight loss over 285 °C was probably due to the decomposition of CNCs. Moreover, the total weight losses of the TMX-CNCs and CNCs were about 88.2% and 69.5% in the range of 212–800 °C, indicating that about 18.7% of the TMX was loaded into the CNCs. 

### 3.2. In Vitro Release of TMX 

**Figure 7:**
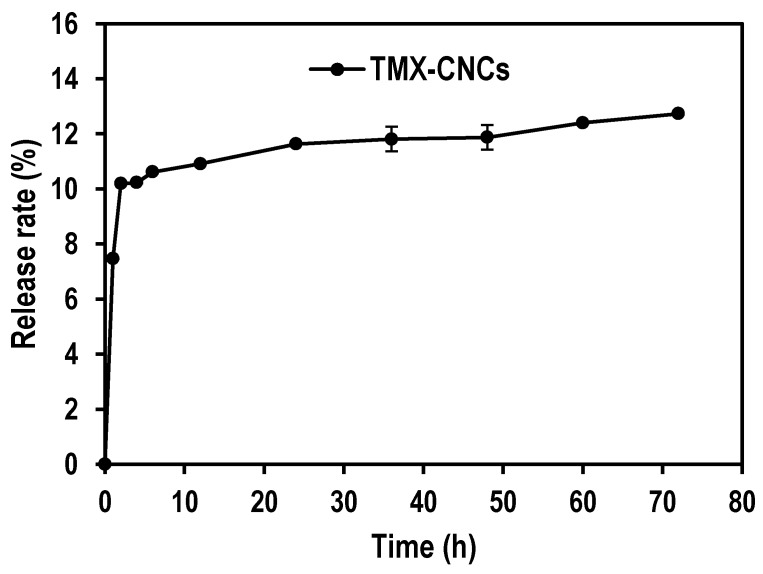
Release rate % of TMX from TMX-loaded CNCs in phosphate buffer solution (pH 7.4). Errors bars indicate standard deviation (*n* = 3).

The release profile of TMX from TMX-CNCs was performed in phosphate buffer saline solution (pH 7.4), as the release media with shaking at a constant rate of 100 rpm at 25 °C. As shown in Figure 7, the release rate of TMX from CNCs was relatively fast during the initial 6 h, and slowed down afterwards with increasing time. The cumulative release amount of TMX-CNCs was 12.73 ± 0.02% after 72 h. The release data demonstrated that there were two stages of TMX release from TMX-CNCs, which could be explained as below. First, the initial rapid release behavior may be due to the presence of TMX close to the surface of CNCs. Second, the hydrogen bonding interaction between TMX molecules and CNCs surface could also be the main parameter that prevents the release. The released TMX concentrations have been not equal to the maximum quantity of TMX-loaded CNCs, because the particles have not been destroyed in the PBS solution. The findings suggest that TMX-CNCs display a slower and good sustained release, which can be expected to improve pesticide-controlled release formulation, and decrease the effective dose via maintaining an adequate concentration against target pests for longer times. Another benefit for CNCs as carrier systems is that they are simple to synthesize, making them an attractive choice for agricultural applications.

### 3.3. Bioassay

In this study, the second instar nymphs of *P. solenopsis* were selected as the model insect to assess the biological activity of TMX-CNCs in comparison to the commercial formulation (25% WDG) and TMX technical (TC) by the leaf dipping method. For each formulation, the LC_50_ value was calculated and expressed in terms of the concentration (µg/mL) for three days. The bioassay results of insecticidal activity for TMX-CNCs, technical, and the conventional formulation against *P. solenopsis* are summarized in Table 2. As calculated from bioassay results of insecticidal activity for TMX-CNCs, technical, and the commercial formulation, their LC_50_ values were 0.25, 0.28, and 0.55 µg/mL after 72 h, respectively. Compared with the LC_50_ values at 24, 48, and 72 h, LC_50_ was shown to be significantly lower for TMX-CNCs than for the technical and commercial formulation at all periods of exposure. Therefore, the toxicity of TMX-CNCs was 1.1 and 2.2 times that of the technical and commercial formulation after 72 h of exposure, respectively, which suggested that the biological activity was considerably improved through using CNCs as the carrier, and TMX-CNCs have significantly better insecticidal toxicity than that of the technical and commercial formulation. Moreover, TMX NF can save the energy and manpower through decreasing the number of pesticide applications needed, pesticide dosage, and minimize the non-target effects compared to traditional pesticide. The high efficacy of TMX nanoformulation might be due to the small particle size and large specific surface area, which can increase the penetration and absorption of the active ingredients by the pest [52,53]. These results agreed with the previous study by Saini et al., who reported that the bioavailability of pyridalyl nanosuspension against *H. armigera* was more effective than the technical product and commercial formulation, and the increased toxicity of nanosized formulation on larvae probably owed to an increased penetration of pyridalyl in the larval body [54]. In another study, Wang et al. [55] stated that the LCNS had a better insecticidal effect than conventional suspension formulations against the Mustard aphid. We recently prepared emamectin benzoate (EMB)-loaded CNCs and found that the biological activity of EMB + CNCs was 6.2-fold more efficient than the commercial EMB EC formulation against *P. solenopsis*. [36] 

## 4. Conclusions

In summary, the nanoformulation of TMX (TMX-CNCs) with biodegradable CNCs as a carrier was successfully synthesized by an emulsion solvent evaporation technique. Loading efficiency and entrapment efficiency of TMX were 18.7% and 83.7 ± 1.8%, respectively. The synthesized TMX-CNCs had a length of 85–214 nm and a width of 7–14 nm with a zeta potential of −23.6 ± 0.3 mV. The FT-IR and TGA analyzes confirmed that the TMX was loaded with CNCs. The TMX-loaded CNCs showed good and sustained release behavior performance. Moreover, TMX-CNCs exhibited better insecticidal activity against cotton mealybug (*P. solenopsis*) than the technical and commercial formulations. The toxicity of TMX-CNCs was 1.1 and 2.2 times that of the technical and commercial formulations after 72 h of exposure. The results of this research demonstrated that the novel nanoformulation could have promising potential for wide applications in agriculture. Therefore, using these smart nano-pesticide formulations, we envision that pesticide losses may be significantly reduced, and that efficiency of use may be improved, which could lead to decreased pesticide dose, number of application times, and negative effects on the environment.

## Figures and Tables

**Table 1:** Dynamic light scattering (DLS)/Zeta potential of CNCs and TMX-loaded CNCs (mean ± SD, n=3).

Sample		Nanoformulation		
	ζ (mV) ^a^	Size (nm)	PdI ^b^	EE (%) ^c^
CNCs	−39.0 ± 1.8	124.5 ± 0.5	0.3 ± 0.0	-
TMX-CNCs	−23.6 ± 0.3	798.0 ± 149	1.0 ± 0.0	83.7 ± 1.8

^a^ Zeta potential, ^b^ Polydispersity Index, ^c^ Entrapment Efficiency.

**Table 2:** Toxicity of TMX formulations against the second instar nymphs of *P. solenopsis*.

Formulation	Time (h)	LC_50_ (95% CL, µg/mL)	Slope ± SE	χ^2^
TMX-CNCs	24	0.70 (0.60–0.85)	3.34 ± 0.60	2.63
	48	0.37 (0.24–0.48)	1.88 ± 0.44	2.35
	72	0.25 (0.11–0.35)	1.75 ± 0.44	2.96
TMX (TC)	24	0.91 (0.51–1.83)	0.89 ± 0.25	0.19
	48	0.44 (0.15–0.78)	0.85 ± 0.25	0.12
	72	0.28 (0.10–0.45)	1.09 ± 0.27	1.83
TMX 25% WDG	24	2.11 (1.60–3.71)	1.80 ± 0.47	1.45
	48	1.02 (0.53–1.47)	1.34 ± 0.42	0.77
	72	0.55 (0.15–0.83)	1.45 ± 0.44	1.12
